# Emergency department crowding and mortality: an observational multicenter study in Sweden

**DOI:** 10.3389/fpubh.2023.1198188

**Published:** 2023-07-25

**Authors:** Jens Wretborn, Daniel B. Wilhelms, Ulf Ekelund

**Affiliations:** ^1^Department of Emergency Medicine, Faculty of Health Sciences, Linköping University, Linköping, Sweden; ^2^Department of Biomedical and Clinical Sciences, Faculty of Health Sciences, Linköping University, Linköping, Sweden; ^3^Department of Clinical Sciences Lund, Emergency Medicine, Faculty of Medicine, Lund University, Lund, Sweden

**Keywords:** emergency department, crowding, overcrowding, occupancy, access block

## Abstract

**Background:**

Emergency department (ED) crowding is a serious problem worldwide causing decreased quality of care. It is reasonable to assume that the negative effects of crowding are at least partially due to high staff workload, but previous crowding metrics based on high workload have not been generalisable to Swedish EDs and have not been associated with increased mortality, in contrast to, e.g., occupancy rate. We recently derived and validated the modified Skåne Emergency Department Assessment of Patient Load model (mSEAL) that measures crowding based on staff workload in Swedish EDs, but its ability to identify situations with increased mortality is unclear. In this study, we aimed to investigate the association between ED crowding measured by mSEAL model, or occupancy rate, and mortality.

**Methods:**

All ED patients from 2017-01-01 to 2017-06-30 from two regional healthcare systems (Skåne and Östergötland Counties with a combined population of approximately 1.8 million) in Sweden were included. Exposure was ED- and hour-adjusted mSEAL or occupancy rate. Primary outcome was mortality within 7 days of ED arrival, with one-day and 30-day mortality as secondary outcomes. We used Cox regression hazard ratio (HR) adjusted for age, sex, arrival by ambulance, hospital admission and chief complaint.

**Results:**

We included a total of 122,893 patients with 168,900 visits to the six participating EDs. Arriving at an hour with a mSEAL score above the 95th percentile for that ED and hour of day was associated with an non-significant HR for death at 7 days of 1.04 (95% CI 0.96–1.13). For one- and 30-day mortality the HR was non-significant at 1.03 (95% CI 0.9–1.18) and 1.03 (95% CI 0.97–1.09). Similarly, occupancy rate above the 95th percentile with a HR of 1.04 (95% CI 0.9–1.19), 1.03 (95%CI 0.95–1.13) and 1.04 (95% CI 0.98–1.11) for one-, 7- and 30-day mortality, respectively.

**Conclusion:**

In this multicenter study in Sweden, ED crowding measured by mSEAL or occupancy rate was not associated with a significant increase in short-term mortality.

## Introduction

Emergency Department (ED) crowding occurs when the demand for ED care is higher than the available resources, and has been associated with increased morbidity, mortality and decreased quality of ED care (1, 2). Historically, crowding has been a minor problem in Swedish EDs (3), but recent reports have found an association between increased length of stay or high occupancy rate and increased mortality (2, 4, 5). There are several combined measures based on staff assessment of crowding, like the national emergency department overcrowding score (NEDOCS), international crowding measure in emergency departments (ICMED) and Emergency Department Work Index (EDWIN). However, these models have not been evaluated against patient mortality and are not readily generalisable to Swedish EDs due to differences in ED operations between health care systems (6).

We have previously derived and validated the modified Skåne Emergency Department Assessment of Patient Load model (mSEAL) to measure crowding based on workload in Swedish EDs (6). The model includes the variables *Patient Hours* and *Time to Physician. Patient hours* is defined as the sum of time in hours that all patients spent in the ED during the previous hour, divided by the ED census during the same hour. *Time to Physician* is the average time in hours, from registration at the ED to first physician contact, for patients waiting for a physician during the previous hour. The mSEAL score is calculated as 1.485 + (9.715*Patient Hours) + (0.177*Time to Physician), with results ranging from 1 (no crowding) to 6 (extreme crowding). The mSEAL model correlated well with staff-assessed crowding in the derivation and validation study, with test characteristics comparable to that of occupancy rate (6). However, the model’s ability to identify situations where crowding affects patient outcomes is still unclear.

Occupancy rate (OR) has been promoted as an easy-to-use metric for crowding that performs equal to more complex models (7), like the mSEAL, but its ability to predict mortality related to crowding has varied in recent studies. Jo et al. (8) and Af Ugglas et al. (2) found an association between high OR and increased mortality in two Korean hospitals and in a Swedish county, respectively. In contrast, Jones et al. (9) and Derose et al. (10) found no association between OR and mortality in large studies from New Zealand and the US, respectively.

It is reasonable to assume that a substantial proportion of the negative effects of crowding are mediated through high workload, and that a metric based on staff perception may provide additional information compared to operational measures like OR. The aim of the present study was to investigate if ED crowding, as measured by mSEAL based on staff assessment or OR, is associated with increased all-cause short-term mortality.

## Methods

### Study design and setting

This was a retrospective observational cross-sectional study of patients at six EDs in two regions in Sweden (Skåne and Östergötland Counties with a combined population of approximately 1.8 million at the time of the study), including three academic tertiary care centers, two urban community hospitals and one community hospital. EDs in Sweden are publicly funded and provide care for all with a small co-payment. All patients visiting any of the EDs from 2017-01-01 to 2017-06-30 were included in the analysis.

### Measurements

Data on age (0–18, 19–39, 40–59, 60–79, >80 years), sex (male/female), hospital admission (yes/no), arrival by ambulance, triage acuity and visit timestamps were extracted for each patient from the electronic health records at each site. All data were registered by ED providers as part of routine ED care. The only generally accepted quality indicator metrics in Sweden is ED length of stay and time to physician (11). However, no reimbursements are associated with these metrics so any systematic influence on registration is unlikely. Mortality data were retrieved from the Swedish national ED quality registry (SVAR) (12), censored at 30 days. SVAR retrieves mortality data from the national population registry and includes all deaths of Swedish citizens. Acuity was defined as the first triage category assigned on ED according to the RETTS triage system (1–4, 1 is the highest acuity) used by all EDs (13) in this study and by a majority of EDs in Sweden (14). Three EDs used an optional 5th category which were combined with category 4 in the analysis. In the regression model, acuity was inverted so that 1 represented the lowest acuity. To adjust for chief complaints, which may affect mortality, the ten complaints with the highest absolute 30-day mortality were each added as a category to the analysis, with the remaining ones categorized as *other*.

### Outcomes

The primary outcome was 7-day all-cause mortality for both admitted and discharged ED patients measured as cox proportional hazard ratios. We hypothesized that the negative effects of ED crowding would have the greatest impact on short-term mortality, which is more likely to be related to the quality of ED care. Secondary outcomes wereone- and 30-day mortality. Outcomes were analyzed unadjusted and adjusted for age, sex, acuity, arrival by ambulance, hospital admission and chief complaint.

### Exposures

The exposure was crowding, defined as a mSEAL score at the hour of arrival for each patient. Additional analyzes were performed using the alternative exposures of occupancy rate (occupancy rate; number of present ED patients divided by treatment beds) at patient arrival. To account for baseline differences in occupancy rate between the included study sites (15) and intra-day variations (2, 12, 16), each crowding metric was stratified by ED and arrival hour. The exposure variable for each patient was categorized by quantile (<85%, 85–90%, 90–95, >95%) since we hypothesized that the effects of crowding on mortality are non-linear which is supported by previous studies by Af Ugglas et al. (2) and McCusker et al. (17). The <85% category was used as reference for each exposure group. To account for repeated visits, which may be more prevalent in patients who die, results are reported with and without left-truncation of data. Left truncation removes all subsequent ED visits until censoring for each index visit. Hours when the mSEAL score could not be calculated, often due to few patients or when no patients had been waiting for a physician, were denoted missing and excluded from the regression model.

### Analysis

Mortality risk was calculated using a cox proportional hazard model adjusted for age, sex, hospital admission, acuity, chief complaint and EMS arrival. Based on an estimated 7-day mortality of 0.5%, 20,000 visits were needed to identify an 0.4% absolute difference in mortality with a power of 0.8 and alpha risk of 0.05, using Fisher’s exact test (18, 19). To allow for subgroup analyzes and adjustments for confounding factors, we chose a 6-month inclusion period which would correspond to approximately 150,000 visits. A point estimate with 95% confidence intervals not including 1.0 and a value of p less than 0.05 were defined as statistically significant. Data were imported into Pandas (v 0.23) (20) and analyzed with Python using the Scipy library (v 1.17) (21) and the lifelines library for survival analysis (22). This study was carried out in accordance with The Declaration of Helsinki (23) and approved by the regional ethics review board at Lund (permit number 2016/69).

## Results

**Figure 1:**
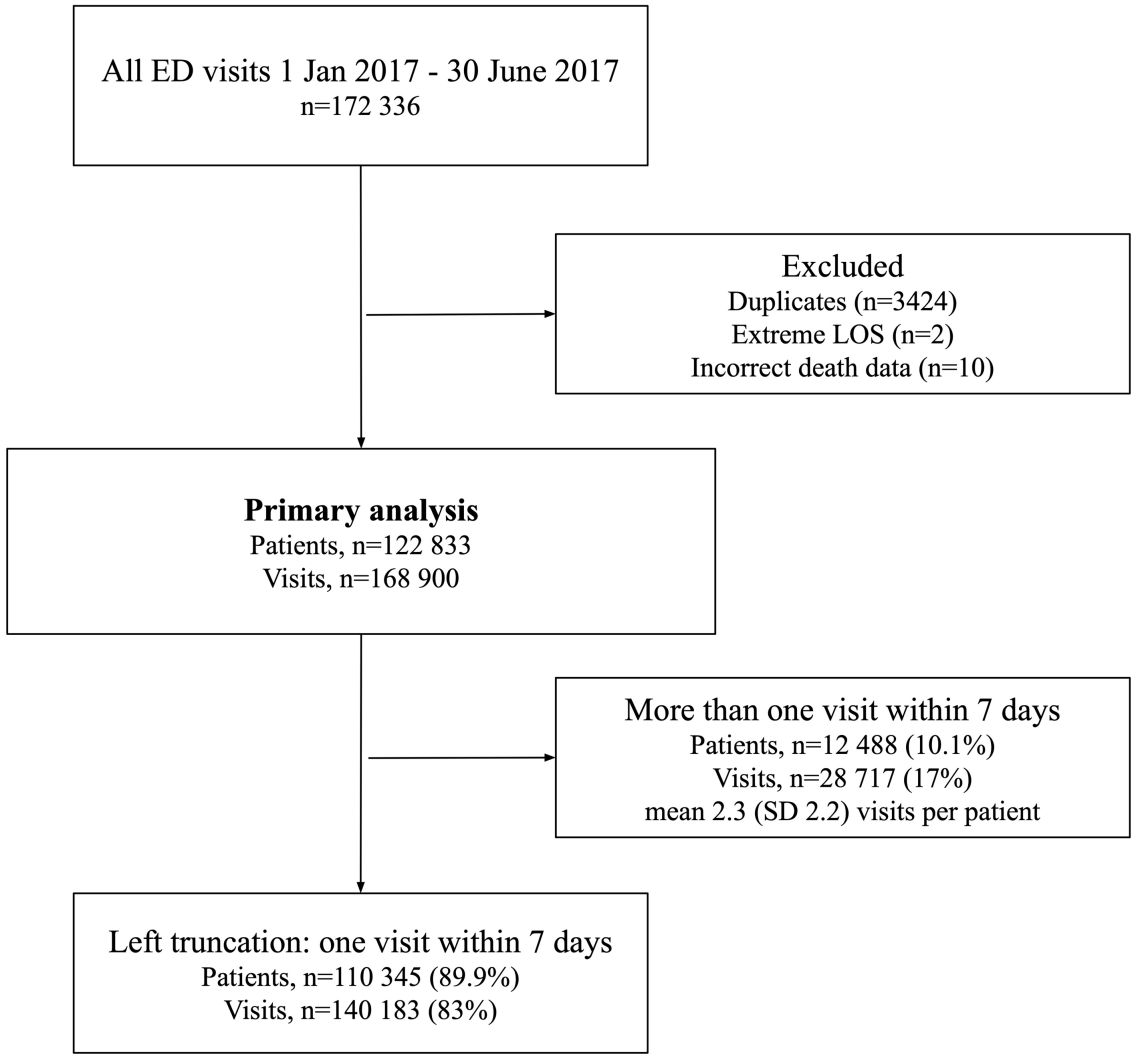
Flowchart of included visits and patients.

A total of 172,336 visits were made to the study EDs during the study period. After excluding duplicates (*n* = 3,424), very long lengths of stay (LOS) likely to be incorrect (>3 days, *n* = 2) and incorrect mortality data (*n* = 10), 168,900 visits made by 122,833 patients were included in the final analysis (Figure 1). There were considerable differences in hospital admissions, 30 day mortality and LOS between the included EDs (Table 1).

**Table 1:** Characteristics of the EDs included in this study.

Type	Helsingborg	Linköping	Lund	Malmö	Motala	Norrköping
	Urban Community	Academic Tertiary Center	Academic Tertiary Center	Academic Tertiary Center	Rural Community	Urban Community
ED beds	52	48	35	72	16	29
Annual visits	88,486	40,502	67,458	79,412	21,194	40,748
Hospital Admissions	22%	21%	27%	28%	19%	24%
Mortality^a^						
1-day	0.33%	0.29%	0.37%	0.45%	0.28%	0.25%
7-day	0.77%	0.72%	0.85%	1.13%	0.95%	0.86%
30-day	1.72%	1.47%	2.00%	2.45%	2.05%	1.84%
Length of stay						
Mean (SD)	04:18 (04:52)	03:40 (02:23)	05:02 (03:57)	04:19 (03:03)	03:11 (02:04)	03:20 (02:05)
Median (IQR)	03:00 (01:13–05:29)	03:19 (01:50–05:05)	04:10 (02:22–06:39)	03:43 (02:05–05:53)	02:47 (01:41–04:21)	02:59 (01:50–04:28)

a Admitted and discharged patients combined.

Table 2 shows that LOS and unadjusted mortality for all three follow-up periods seemed to increase with increased crowding. The reference group had higher mortality than the lowest crowding group and was similar to the >90% groups. Mortality was generally higher in the reference group compared to patients arriving at hours when the mSEAL score could not be calculated (missing).

**Table 2:** Outcome characteristics stratified by mSEAL crowding category.

	<85%	85–90%	90–95%	95–100%	Missing
N	143,041	8,394	8,527	7,974	964
Arrival by ambulance	25.2%	25.4%	24.8%	24.6%	22.5%
Female	49.8%	50.4%	49.7%	49.6%	49.9%
Mortality (*n*)^a^					
1-day	0.35% (503)	0.27% (23)	0.34% (29)	0.41% (33)	0.21% (2)
7-day	0.89% (1277)	0.73% (61)	0.88% (75)	1.03% (82)	0.31% (3)
30-day	1.97% (2821)	1.6% (134)	1.97% (168)	2.03% (162)	1.14% (11)
LOS					
Mean (SD)	04:05 (03:32)	04:40 (04:05)	04:41 (04:05)	04:58 (04:22)	04:39 (04:05)
Median(IQR)	03:19 (01:46–05:25)	03:47 (02:01–06:06)	03:49 (02:05–06:04)	04:01 (02:09–06:25)	03:46 (02:05–06:04)

a Admitted and discharged patients combined, unadjusted.

The Cox proportional hazard model adjusted for age, sex, hospital admission, acuity, chief complaint and EMS arrival is described in Table 3. High levels of crowding, defined as a mSEAL score over the 95th percentile for ED and hour of day, was associated with a non-significant increase in HR for 7-day mortality of 1.04 (95% CI 0.96–1.13). Similarly, an occupancy rate above the 95th percentile at arrival was associated with a non-significant increase HR for 7-day mortality of 1.03 (0.95–1.13). The HR for one- and 30-day mortality for mSEAL and occupancy rate above the 95th percentile were also increased, but all results were not statistically significant with confidence intervals included one. When left-truncating the data, i.e., removing all subsequent visits by the patients during the follow-up period, the statistical power decreased and 95% confidence intervals widened for all models (Supplementary Tables S1). In our cohort, 4.6% of patients who died within 7 days of a visit had >1 visit during these 7 days. Of patients who did not die, 7.5% had >1 visit within 7 days.

**Table 3:** Cox proportional hazard ratio for all cause mortality at each crowding exposure.

	1-day	7-day	30-day
mSEAL			
85–90%	1.05 (0.67–1.63)	0.93 (0.71–1.22)	0.85 (0.71–1.02)
90–95%	0.93 (0.76–1.13)	1.01 (0.9–1.14)	1.01 (0.93–1.1)
>95%	1.03 (0.9–1.18)	1.04 (0.96–1.13)	1.03 (0.97–1.09)
Occupancy rate			
85–90%	0.98 (0.68–1.42)	0.92 (0.72–1.17)	0.92 (0.78–1.08)
90–95%	0.9 (0.71–1.14)	0.92 (0.79–1.06)	0.92 (0.84–1.01)
>95%	1.04 (0.9–1.19)	1.03 (0.95–1.13)	1.04 (0.98–1.11)

Models adjusted by: age, sex, EMS arrival, acuity and admission to hospital with 95% confidence intervals.

There were large differences in average mSEAL score between the study EDs for each exposure group, as shown in Table 4. The highest average reference and 95th percentile scores (3.79 and 5.17) were observed at Lund, and the lowest at Motala (3.03) and Norrköping (4.25), respectively. Similarly, there were large differences in occupancy rate at the 85th and 95th percentile for each hour of day and ED where the 95th percentile occupancy rate was consistently 2–4 times higher in the ED with the highest occupancy rate (Lund) compared to the ED with the lowest occupancy rate (Linköping) (Supplementary Tables S2, S3).

**Table 4:** mSEAL average score (SD) by hospital and crowding group.

	Helsingborg	Linköping	Lund	Malmö	Motala	Norrköping
<85% (reference)	3.60 (0.63)	3.36 (0.91)	3.79 (0.72)	3.55 (0.67)	3.03 (0.78)	3.11 (0.73)
85–90%	4.40 (0.55)	4.07 (0.98)	4.61 (0.67)	4.24 (0.64)	3.81 (0.90)	3.73 (0.82)
90–95%	4.60 (0.53)	4.26 (1.03)	4.81 (0.71)	4.40 (0.67)	3.99 (0.94)	3.88 (0.87)
95–100%	5.09 (0.59)	4.86 (1.25)	5.17 (0.77)	4.70 (0.74)	4.40 (1.04)	4.25 (0.90)

## Discussion

In this study, crowding defined as an mSEAL score above the 95th percentile had no statistically significant association with increased HR for 7-day mortality adjusted for age, sex, arrival by EMS, acuity, chief complaint and admission to hospital, compared with non-crowding (score < 85th percentile). There were positive hazard ratios for death in the highest crowding group (>95th percentile) for both mSEAL and occupancy rate, but the 95% CI included one, and the results were therefore not significant at the *p* < 0.05 level.

Crowding occurs when the demand for ED care is higher than the available resources with negative effects for patients and providers (24). Although the definition is universal, the extent of the problem and its consequences may vary between institutions, being more prominent in urban and academic EDs (25, 26). A recent study by Af Ugglas et al. (27) found variable association between crowding and mortality within Sweden and a previous study by our group showed considerable differences in resource availability in Sweden, specifically treatment beds and staffing in relation to patient volume (15). Our study included a mix of rural, urban and academic centers which may contribute to the lack of association between crowding and mortality. Although initial analysis indicated variation in mortality between sites, the differences were small given the limited overall absolute mortality and further subgroup analysis was not performed.

The average mSEAL score of the different crowding groups varied substantially between the EDs; Lund ED had the highest average score of 5.17 at the 95th percentile group and Norrköping ED the lowest at 4.25. There was also a large variation in baseline occupancy rate (Supplementary Table S2). Previous studies from our group and others (15, 28–30) have shown that crowding may indeed vary between EDs and that scores need to be adjusted to the individual site. In this study, we also found large intra-day variations at all EDs for both mSEAL scores and occupancy rate (Supplementary Tables S2, S3). By stratifying the scores by ED and hour of day we accounted for these differences, making them comparable between EDs and likely interchangeable as markers of crowding.

When left-truncating the data (counting only the first visit during the follow-up period), the HR point estimates decreased and the uncertainty of the results increased, likely due to less statistical power. Truncation will potentially remove bias caused by multiple ED visits before death that may systematically skew the results if patients that are more likely to die visit the ED more often than other patients. This has been shown during the last year of life (31–33), but is unknown for short-term follow-up. It was not the case in our study where patients with repeated visits within the follow-up period had lower mortality compared to the cohort, limiting the possible confounding. Furthermore, truncation may increase the risk of missing a true crowding effect on mortality since crowding may appear at an excluded ED visit. ED visit data were left truncated in the study by Af Ugglas et al. (2), but not in the study by Jones et al. (9).

### Limitations

There are several limitations to this study, the first one being the lack of statistical power and a risk of a type II statistical error. At the time of power calculation there were only two previous studies available on the association between crowding and mortality, with effect sizes between 5 and 34%, which contributed to the uncertainty regarding the correct effect size (18, 19). The mSEAL and OR effect estimates showed the expected trends towards increased HR which is consistent with the findings of Af Ugglas et al. (2) in a larger study with comparable methods. Furthermore, several previous studies have found an association between ED crowding and mortality supporting an underlying association (2, 9, 17, 18, 34).

This was a retrospective observational study and as such, it can only demonstrate association and not causation. We lacked data to control for previous medical history of the patients with the Charlson comorbidity index, as initially planned, which may explain some of the observed effect on mortality. However, we were able to control for all significant confounders found in a similar study where the addition of previous medical data did not alter the results (2). There may be additional confounding variables like exogenous events (e.g., EHR malfunction, seasonal effects), that may affect our results (35, 36).

## Conclusion

In this multicenter study in Sweden, high ED crowding measured by mSEAL or occupancy rate was not significantly associated with an increase in short-term mortality.

## Data availability statement

The raw data supporting the conclusions of this article will be made available by the authors, without undue reservation.

## Ethics statement

The studies involving human participants were reviewed and approved by Ethics committee of Lund University, permit number 2016/69. The ethics committee waived the requirement of written informed consent for participation.

## Author contributions

JW, DW, and UE conceived the study and designed the trial. UE obtained the ethical permit. JW coordinated data collection and managed the data, with the assistance of DW and UE. DW and UE obtained the research funding. JW drafted the manuscript and takes responsibility for the paper as a whole. All authors contributed to the article and approved the submitted version.

## Funding

This work was supported by two grants from Region Östergötland to author DW (LIO-532001 and LIO-700271), and from Region Skåne to author UE. The funding bodies had no role in study design, data collection, data analysis or writing of the manuscript.

## Conflict of interest

The authors declare that the research was conducted in the absence of any commercial or financial relationships that could be construed as a potential conflict of interest.

## Publisher’s note

All claims expressed in this article are solely those of the authors and do not necessarily represent those of their affiliated organizations, or those of the publisher, the editors and the reviewers. Any product that may be evaluated in this article, or claim that may be made by its manufacturer, is not guaranteed or endorsed by the publisher.

## Abbreviations

ED: Emergency Department
NEDOCS: National Emergency Department Overcrowding Score
ICMED: International Crowding Metric in Emergency Department
EDWIN: Emergency Department Work Index
mSEAL: modified Swedish Emergency Department Assessment of Patient Load
EMS: Emergency Medical Services
LOS: Length of Stay
HR: Hazard Ratio
