# Supplementary material for: Emergency department crowding and mortality: an observational multicenter study in Sweden
Source: Front Public Health. 2023 Jul 25;11:1198188. doi: 10.3389/fpubh.2023.1198188 (PMC10407086; doi:10.3389/fpubh.2023.1198188)
Supplement: Supplementary file 1 [file Table_1.pdf]

Table S1. Cox proportional hazard ratio for each crowding exposure and all cause mortality with 95% confidence interval and left truncation of data with <85 th percentile as reference

	1-day	7-day	30-day
mSEAL			
85-90%	1.02 (0.65 - 1.6)	0.94 (0.71 - 1.25)	0.86 (0.69 - 1.06)
90-95%	0.93 (0.77 - 1.13)	0.98 (0.86 - 1.11)	1.01 (0.92 - 1.11)
>95%	1.02 (0.89 - 1.17)	1.03 (0.95 - 1.12)	1 (0.94 - 1.07)
Occupancy Rate			
85-90%	0.95 (0.65 - 1.38)	0.91 (0.7 - 1.17)	0.94 (0.79 - 1.14)
90-95%	0.91 (0.72 - 1.15)	0.92 (0.79 - 1.07)	0.89 (0.8 - 1)
>95%	1.03 (0.89 - 1.18)	1.02 (0.93 - 1.11)	1.03 (0.96 - 1.1)

Table S2. 95th percentile value (85th percentile reference) for mSEAL at each hour and ED

mSEAL	Helsingborg	Linköping	Lund	Malmö	Motala	Norrköping
0:00	4.67 (4.33)	2.58 (2.38)	4.82 (4.46)	4.6 (4.34)	2.53 (2.27)	2.48 (2.26)
1:00	4.47 (4.07)	2.22 (1.85)	4.53 (4.21)	4.5 (4.1)	2.02 (1.79)	2 (1.8)
2:00	4.17 (3.85)	2.44 (2.16)	4.3 (3.93)	4.16 (3.9)	2.07 (1.94)	2.17 (2.03)
3:00	3.99 (3.64)	2.51 (2.27)	3.94 (3.71)	3.89 (3.66)	2.12 (1.98)	2.29 (2.19)
4:00	3.78 (3.42)	2.6 (2.3)	3.79 (3.58)	3.67 (3.41)	2.17 (2.02)	2.4 (2.22)
5:00	3.57 (3.26)	2.57 (2.29)	3.63 (3.4)	3.39 (3.2)	2.16 (2.01)	2.46 (2.23)
6:00	3.45 (3.12)	2.42 (2.28)	3.49 (3.29)	3.23 (3.01)	2.18 (2.02)	2.4 (2.22)
7:00	3.33 (3.01)	2.49 (2.22)	3.41 (3.12)	3.1 (2.88)	2.25 (2.04)	2.4 (2.2)
8:00	3.4 (3.04)	2.58 (2.3)	3.37 (3.15)	3.14 (2.9)	2.41 (2.22)	2.48 (2.3)
9:00	3.63 (3.25)	2.87 (2.62)	3.61 (3.3)	3.22 (2.98)	2.72 (2.55)	2.75 (2.54)
10:00	3.94 (3.53)	3.28 (3.02)	3.92 (3.62)	3.26 (3.07)	3.12 (2.89)	3.09 (2.9)
11:00	4.31 (3.85)	3.74 (3.53)	4.27 (3.92)	3.66 (3.45)	3.8 (3.38)	3.53 (3.3)
12:00	4.6 (4.21)	4.26 (3.99)	4.64 (4.25)	4.02 (3.75)	4.26 (3.86)	3.86 (3.66)
13:00	5.01 (4.57)	4.68 (4.38)	4.99 (4.65)	4.46 (4.14)	4.47 (4.22)	4.33 (4.01)
14:00	5.19 (4.77)	5.2 (4.79)	5.24 (4.9)	4.71 (4.5)	4.86 (4.48)	4.65 (4.36)
15:00	5.15 (4.77)	5.35 (4.93)	5.41 (4.96)	5.01 (4.56)	5.05 (4.54)	4.8 (4.47)
16:00	5.24 (4.8)	5.29 (4.98)	5.5 (5)	5.11 (4.77)	5.01 (4.53)	4.84 (4.42)
17:00	5.21 (4.79)	5.35 (4.93)	5.68 (5.06)	5.2 (4.8)	4.92 (4.56)	4.85 (4.39)
18:00	5.2 (4.67)	5.36 (4.93)	5.7 (5.14)	5.29 (4.86)	4.74 (4.49)	4.81 (4.4)
19:00	5.02 (4.59)	5.17 (4.77)	5.63 (5.13)	5.2 (4.77)	4.59 (4.2)	4.69 (4.28)
20:00	4.81 (4.5)	4.94 (4.51)	5.54 (5.08)	4.95 (4.7)	4.31 (3.96)	4.38 (3.95)
21:00	4.79 (4.41)	4.5 (4.16)	5.34 (4.95)	4.85 (4.59)	4.15 (3.7)	4 (3.72)
22:00	4.78 (4.44)	4.01 (3.74)	5.08 (4.78)	4.73 (4.45)	3.71 (3.38)	3.66 (3.4)
23:00	4.82 (4.45)	3.32 (3.07)	4.99 (4.64)	4.82 (4.51)	3.1 (2.91)	3.22 (2.93)

Table S3. 95th percentile value (85th percentile reference) for Occupancy at each hour of day and ED

	Helsingborg	Linköping	Lund	Malmö	Motala	Norrköping
0:00	1.54 (1.38)	0.4 (0.31)	1.91 (1.71)	1.01 (0.94)	0.56 (0.48)	0.62 (0.48)
1:00	1.4 (1.23)	0.27 (0.19)	1.71 (1.51)	0.97 (0.86)	0.37 (0.25)	0.34 (0.28)
2:00	1.23 (1.11)	0.25 (0.19)	1.54 (1.37)	0.83 (0.76)	0.25 (0.19)	0.34 (0.24)
3:00	1.1 (1)	0.31 (0.21)	1.29 (1.2)	0.74 (0.68)	0.31 (0.25)	0.38 (0.31)
4:00	1.02 (0.9)	0.31 (0.21)	1.23 (1.06)	0.67 (0.6)	0.31 (0.25)	0.41 (0.34)
5:00	0.94 (0.81)	0.29 (0.23)	1.08 (0.97)	0.56 (0.5)	0.31 (0.25)	0.48 (0.34)
6:00	0.87 (0.75)	0.25 (0.21)	0.97 (0.86)	0.5 (0.44)	0.31 (0.25)	0.41 (0.34)
7:00	0.87 (0.71)	0.25 (0.21)	0.94 (0.83)	0.48 (0.41)	0.31 (0.25)	0.41 (0.31)
8:00	0.9 (0.75)	0.29 (0.25)	1 (0.86)	0.46 (0.39)	0.44 (0.38)	0.48 (0.38)
9:00	1.04 (0.88)	0.4 (0.33)	1.2 (1.05)	0.5 (0.44)	0.63 (0.56)	0.59 (0.52)
10:00	1.29 (1.07)	0.52 (0.46)	1.48 (1.31)	0.63 (0.57)	0.88 (0.81)	0.86 (0.72)
11:00	1.48 (1.27)	0.65 (0.6)	1.77 (1.6)	0.79 (0.69)	1.25 (1.06)	1.1 (0.97)
12:00	1.63 (1.48)	0.81 (0.75)	2.03 (1.8)	0.91 (0.82)	1.44 (1.25)	1.34 (1.17)
13:00	1.79 (1.62)	0.96 (0.85)	2.17 (1.97)	1.05 (0.93)	1.56 (1.43)	1.48 (1.34)
14:00	1.86 (1.69)	1.06 (0.92)	2.31 (2.14)	1.11 (1.03)	1.69 (1.44)	1.62 (1.48)
15:00	1.94 (1.69)	1.1 (1)	2.43 (2.22)	1.21 (1.08)	1.81 (1.56)	1.66 (1.55)
16:00	1.96 (1.71)	1.1 (1)	2.51 (2.22)	1.22 (1.1)	1.75 (1.5)	1.72 (1.55)
17:00	1.9 (1.75)	1.13 (1)	2.6 (2.25)	1.25 (1.14)	1.81 (1.62)	1.72 (1.52)
18:00	1.88 (1.67)	1.08 (0.98)	2.63 (2.22)	1.29 (1.14)	1.69 (1.56)	1.65 (1.48)
19:00	1.85 (1.63)	1.04 (0.96)	2.57 (2.29)	1.22 (1.13)	1.56 (1.38)	1.65 (1.41)
20:00	1.73 (1.57)	0.96 (0.88)	2.48 (2.22)	1.19 (1.1)	1.5 (1.3)	1.45 (1.31)
21:00	1.67 (1.52)	0.85 (0.77)	2.28 (2.11)	1.15 (1.07)	1.38 (1.18)	1.28 (1.17)
22:00	1.67 (1.48)	0.73 (0.65)	2.14 (1.97)	1.08 (1.03)	1.13 (0.94)	1.17 (0.99)
23:00	1.61 (1.44)	0.58 (0.5)	2.08 (1.88)	1.08 (1.01)	0.94 (0.81)	0.93 (0.76)
